# Electroencephalogram of Age-Dependent Epileptic Encephalopathies in Infancy and Early Childhood

**DOI:** 10.1155/2013/743203

**Published:** 2013-08-19

**Authors:** Lily C. Wong-Kisiel, Katherine Nickels

**Affiliations:** Division of Child and Adolescent Neurology, Department of Neurology, Mayo Clinic College of Medicine, 200 First St. SW, Rochester, MN 55905, USA

## Abstract

Epileptic encephalopathy syndromes are disorders in which the epileptiform abnormalities are thought to contribute to a progressive cerebral dysfunction. Characteristic electroencephalogram findings have an important diagnostic value in classification of epileptic encephalopathy syndromes. In this paper, we focus on electroencephalogram findings of childhood epileptic encephalopathy syndromes and provide sample illustrations.

## 1. Introduction

Epilepsy electroclinical syndromes have characteristic seizure semiology, frequency, duration, inciting factors, and age of seizure onset and are often associated with specific electroencephalogram (EEG) findings. Epileptic encephalopathies are syndromes in which the epileptiform abnormalities are thought to contribute to a progressive cerebral dysfunction. The ictal and interictal EEG patterns can help define the electroclinical syndromes, identify potential etiologies, and guide treatment. A detailed description of each genetic etiology is beyond the scope of this review. This review focuses on the neurophysiological features, including variant patterns relevant to selected etiologies (Table 1).

## 2. Neonatal/Infantile Onset Epilepsy Syndromes

### 2.1. Early Infantile Epileptic Encephalopathy

#### 2.1.1. Clinical Presentation

Ohtahara first recognized early infantile epileptic encephalopathy (EIEE) in neonates who suffered frequent tonic seizures and subsequently developed significant intellectual disability [1]. Later studies of infants with EIEE show average seizure onset within 2-3 months after birth. Brief tonic seizures occur hundreds of times per day, often in clusters of 10 to 40 seizures. Focal seizures present as tonic eye deviation or unilateral clonic contractions. Myoclonic seizures are not a prominent feature [2].

#### 2.1.2. Long-Term Prognosis

 Mortality in EIEE is high during childhood, with 50% dying during the first 2 years of life [2]. Survivors into childhood have pharmacoresistant epilepsy with severe intellectual disability. EIEE evolves to West syndrome during infancy in 75%, Lennox-Gastaut syndrome in older children, or focal epilepsy [3]. 

#### 2.1.3. Etiologies

Cerebral structural abnormality is the most common etiology of EIEE. However, in 13–38% of patients, STXBP1 mutation results in synaptic vesicle protein with impaired neurotransmitter release [4–6]. Children with STXBP1 mutation develop a paroxysmal movement disorder during infancy, which persists beyond the intractable phase of the epilepsy [6].

#### 2.1.4. Interictal EEG

 Suppression burst pattern is a distinct EEG feature during the early phase of EIEE. This EEG pattern is characterized by a suppression phase less than 10 *μ*V lasting from 3 to 5 seconds with paroxysms of high-voltage 150–350 *μ*V delta-theta bursts of spikes, polyspikes, and sharp waves that alternate at regular intervals [1]. The suppression burst pattern continues during both awake and sleep states. This pattern does not imply specific etiology and can also be seen due to hypoxic ischemic encephalopathy, metabolic derangement, medication effects, and hypothermia.

**Figure 1:**
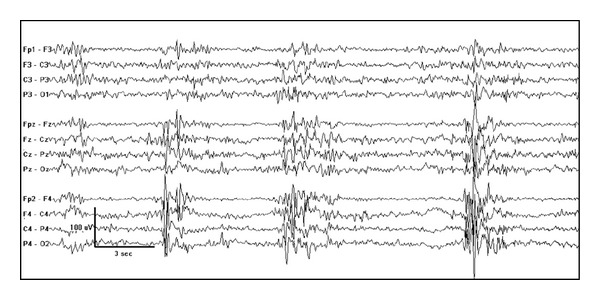
Increased burst amplitude over the right hemisphere in an one-month-old girl with right frontal cortical dysplasia.

For those who survive, EEG wake-sleep differentiation occurs from 40 days to 5 months, with more apparent suppression burst in sleep [7]. During wakefulness, suppression burst pattern evolves into hypsarrhythmia pattern, diffuse slowing, multifocal abnormalities, or pseudoperiodic pattern with improved background organization [8]. Asynchronous attenuations present during wakefulness become more synchronized during sleep. Focal slowing, interictal focal spikes, and increased amplitude bursts may lateralize over the side of the structural abnormality (Figure 1). By one year of life, the EEG shows generalized background slowing and generalized or focal paroxysmal fast activity.

#### 2.1.5. Ictal EEG

The burst activities of the suppression burst pattern coincide with tonic seizures [7]. Focal seizures may start as focal rhythmic spike and wave activity followed by tonic seizures. Ictal discharges for focal seizures show no particular localization and any focus can be involved. 

#### 2.1.6. EEG**  **Variants

Compared to other etiologies, EEGs of affected patients with STXBP1 mutation have longer periods of suppression and bursts [6].

### 2.2. Early Myoclonic Encephalopathy

#### 2.2.1. Clinical Presentation

 Neonates with early myoclonic encephalopathy (EME) have no apparent perinatal complications or insults and present shortly after birth with progressive decreased alertness and hypotonia. Myoclonic seizures are the initial semiology, followed by focal seizures or epileptic spasms. Myoclonic seizures may begin as fragmentary or focal erratic myoclonus involving regions of the face and limbs, at times nearly continuously, and then become more generalized with time. Focal seizures may manifest as gaze deviation or autonomic disturbances. Between 3 and 4 months of age, seizures evolve into epileptic spasms [9].

#### 2.2.2. Long-Term Prognosis

The long-term prognosis of EME is poor. Approximately 50% of infants die in the first year [1]. Those who survive have profound intellectual disability.

#### 2.2.3. Etiologies

Unlike EIEE, metabolic and genetic etiologies are more common than structural abnormalities. EME has classically been associated with nonketotic hyperglycinemia. However, the etiology is unknown in up to 50% and investigations for pyridoxine or pyridoxal-5-phosphate dependency, molybdenum cofactor deficiency, organic aciduria, and amino acidopathies should also be completed [10, 11].

#### 2.2.4. Interictal EEG

**Figure 2:**
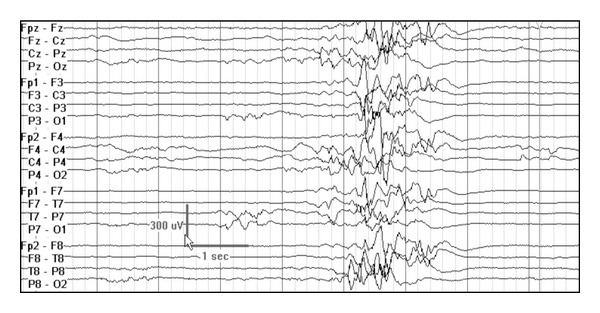
Generalized suppression burst pattern in a newborn with early myoclonic encephalopathy.

The interictal EEG shows the characteristic suppression burst background initially (Figure 2). In contrast with EIEE, the suppression burst pattern in EME is enhanced by sleep with shorter burst duration. The EEG evolves to atypical hypsarrhythmia or multifocal epileptiform discharges with profound slowing of the background EEG activity at age from 3 to 5 months. Hypsarrhythmia pattern may persist for months before a return to burst suppression pattern [12].

#### 2.2.5. Ictal EEG

**Figure 3:**
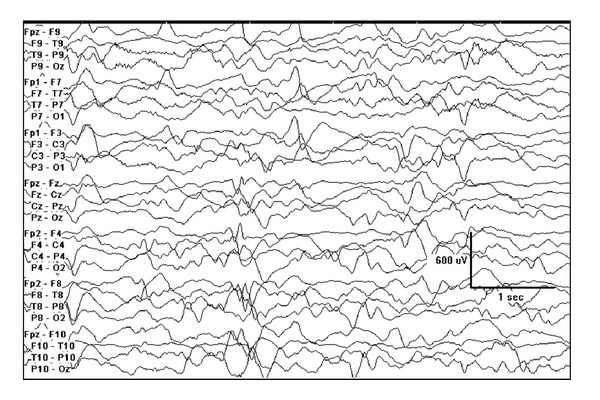
High amplitude poorly organized slow background with multifocal epileptiform discharges and intermittent generalized electrodecrement, consistent with hypsarrhythmia at age 4 months in a child with early myoclonic encephalopathy due to nonketotic hyperglycinemia.

The ictal EEG often shows no correlation with the presenting erratic myoclonia [12] (Figure 3). When focal seizures or epileptic spasms occur, the EEG correlate is not different from focal neonatal seizures seen in nonsyndromic cases.

### 2.3. Migrating Focal Seizures in Infancy

#### 2.3.1. Clinical Presentation

 Migrating focal seizures in infancy is also known as malignant migrating partial epilepsy of infancy, first described by Coppola in 1995 [13]. The mean age of seizure onset is 3 months but can occur as early as the neonatal period [14]. Infants have normal development before seizure onset. At presentation, there are sporadic focal motor seizures with rapid evolution to bilateral convulsive seizures. Autonomic manifestations such as apnea, flushing, or cyanosis often occur. The seizures increase in frequency and become nearly continuous multifocal or bihemispheric seizures. Ictal semiology reflects the affected cortical regions. Epilepsy becomes intractable. Profound psychomotor developmental delay ensues. Seizures of multifocal onset may evolve into epileptic spasms, with or without interictal hypsarrhythmic pattern [13, 15–17].

#### 2.3.2. Long-Term Prognosis

Although better seizure control may be accomplished between 12 and 14 months, neurologic regression and stagnation continue. Children develop cortical visual impairment. Acquired microcephaly or evidence of brain atrophy is seen during followup. There is high mortality before 1 year of age. Illnesses easily trigger clusters of seizures or occasional status epilepticus.

#### 2.3.3. Etiologies

The etiology is heterogeneous and often is unknown. Genetic etiologies include SCN1A mutations, duplication of 16p11.2, homozygous deletion of the PLCB1 gene, and KCNT1 de novo gain-of-function mutation. Deletion in 2q24 was reported in a single patient [18–22].

#### 2.3.4. Interictal EEG

The EEG at seizure onset shows increased background slowing for several months. The background slow waves shift from one hemisphere to the other. Shortly after initial seizures, multifocal discharges emerge and are present during wakefulness and sleep [23]. Epileptiform discharges are most prominent in the temporal and rolandic areas. As seizure frequency subsides after age one year, the EEG shows a low voltage “burnt out” slowing [24].

#### 2.3.5. Ictal EEG

**Figure 4:**
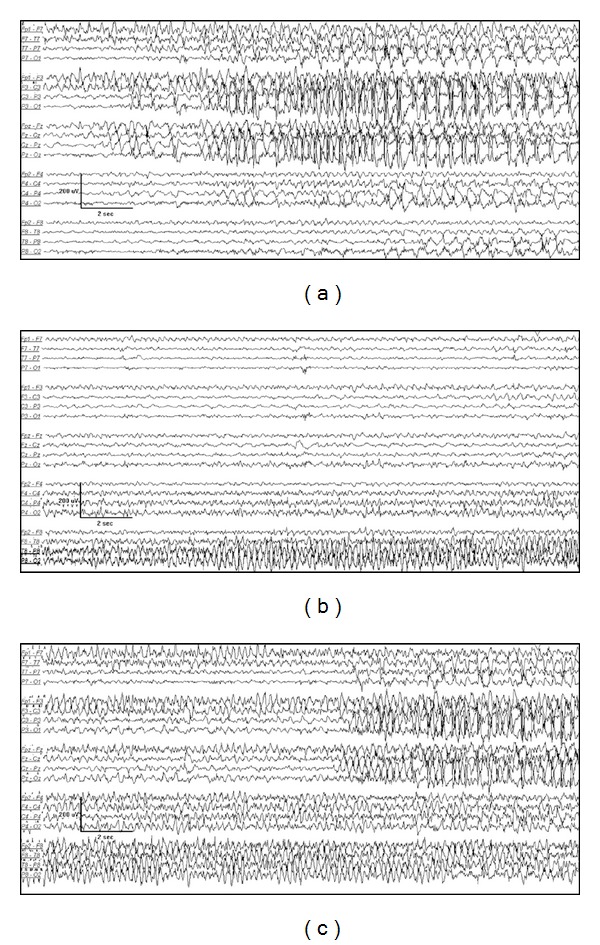
3-month-old female with migrating focal seizures in infancy. (a) Left central onset seizure, (b) right temporal onset seizure 2 minutes later, and (c) independent left central onset seizure with ongoing right temporal lobe seizure 15 minutes later.

Ictal onset shifts in consecutive seizures from one lobe to another and from one hemisphere to another (Figure 4). Although the involved ictal regions vary, the ictal pattern is very similar. Ictal EEG shows rhythmic, monomorphic alpha or theta frequency discharges in a localized cortical area, then expand to contiguous regions, or may develop independently in different areas [13]. Tandem seizures in different noncontiguous brain regions may occur, with one seizure beginning before the waning of the other. Ictal and interictal EEGs almost overlap. The original ictal EEG discharges may persist or fade and be replaced by new patterns, thus producing a very complex multifocal status epilepticus.

### 2.4. West Syndrome

#### 2.4.1. Clinical Presentation

West syndrome refers to the triad of epileptic spasms, intellectual disability, and EEG hypsarrhythmia. Spasm onset is between 3 and 8 months, characterized by brief truncal and neck flexion coinciding with bilateral arm abduction, typically occurring in clusters. Developmental regression occurs.

#### 2.4.2. Long-Term Prognosis

The long-term prognosis of West syndrome is poor, especially if not treated early. Epileptic spasms typically resolve by age 5 years. Other seizure types often emerge and children may develop Lennox-Gastaut syndrome. Learning disorders, intellectual disability, and autistic features are common. Although an etiology is found in the majority of children with epileptic spasms, those of unknown etiology can have a more favorable prognosis if early effective treatment is provided [25]. 

#### 2.4.3. Etiologies

Epileptic spasm etiologies are heterogeneous and include structural, metabolic, and genetic causes. Classically, tuberous sclerosis complex (TSC) has been associated with epileptic spasms and must be excluded at initial evaluation. Asymmetric spasms characterized by unilateral head turn, asymmetric upper extremity flexion or extension, or automatisms suggest lateralized structural abnormality. Congenital central nervous system malformation or TSC accounts for more than half of the cases of epileptic spasms, followed by 15% due to chromosomal abnormalities including trisomy 21 and trisomy 18. Monogenic etiologies including X-linked CDKL5, ARX, and MECP2 are increasingly recognized, particularly in children with seizure onset less than 3 months of age and atypical Rett-like features [25]. Hypermotor-tonic-spasm sequence is not diagnostic for a specific etiology but should raise a suspicion for CDKL5 mutation [26].

#### 2.4.4. Interictal EEG

**Figure 5:**
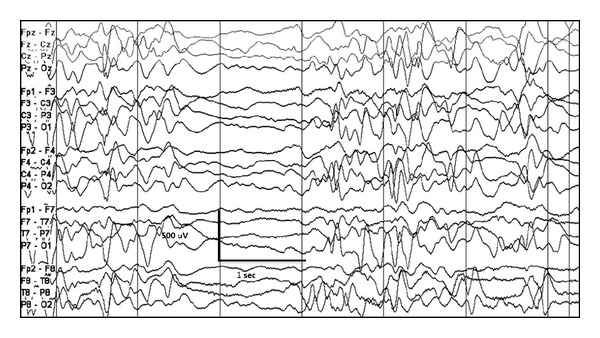
Interictal EEG recording of typical hypsarrhythmia in a two-year-old girl with West syndrome due to genetic mutation.

The classic hypsarrhythmia is an interictal EEG pattern of a poorly organized, high amplitude (500–1000 *μ*V), slow background with accompanying multifocal epileptiform discharges, and generalized electrodecrement (Figure 5). There may be generalized discharges on the interictal recording, but repetitive trains of generalized discharges are uncommon. Lack of hypsarrhythmia in the routine outpatient EEG does not exclude the diagnosis of epileptic spasms. The characteristic EEG features may be present only during non-REM sleep, particularly early in the course of the epilepsy. Prolonged video EEG with recorded spasms and a sleep recording remains an essential diagnostic tool.

#### 2.4.5. Ictal EEG

**Figure 6:**
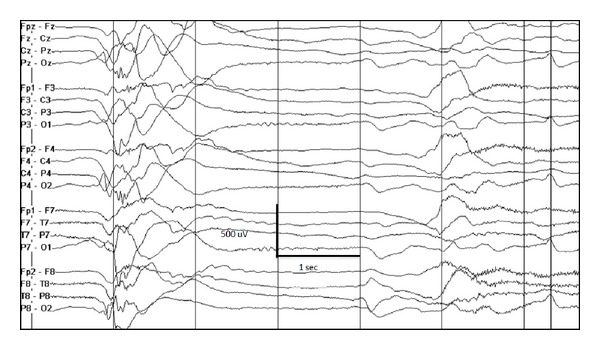
Ictal EEG recording of epileptic spasms in a two-year-old girl with West syndrome due to genetic mutation.

The EEG during the epileptic spasms typically demonstrates a high amplitude generalized sharp wave followed by generalized electrodecrement (Figure 6). The electrodecrement differentiates short spasms from myoclonic seizures.

#### 2.4.6. EEG Variants

**Figure 7:**
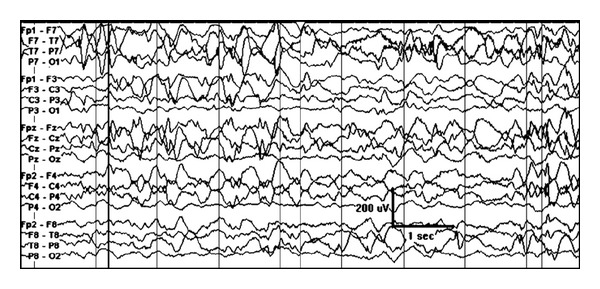
Interictal EEG recording of modified hypsarrhythmia in an 8-month-old child with partially treated epileptic spasms; note the lower amplitude of the background.

**Figure 8:**
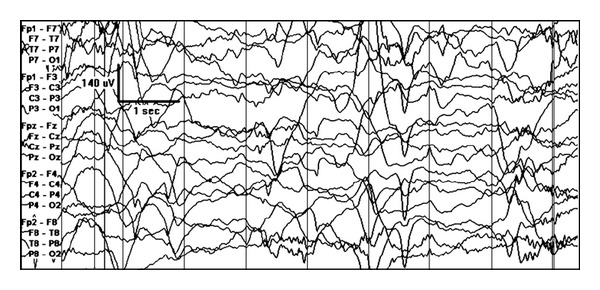
Ictal EEG recording of epileptic spasms with modified hypsarrhythmia.

**Figure 9:**
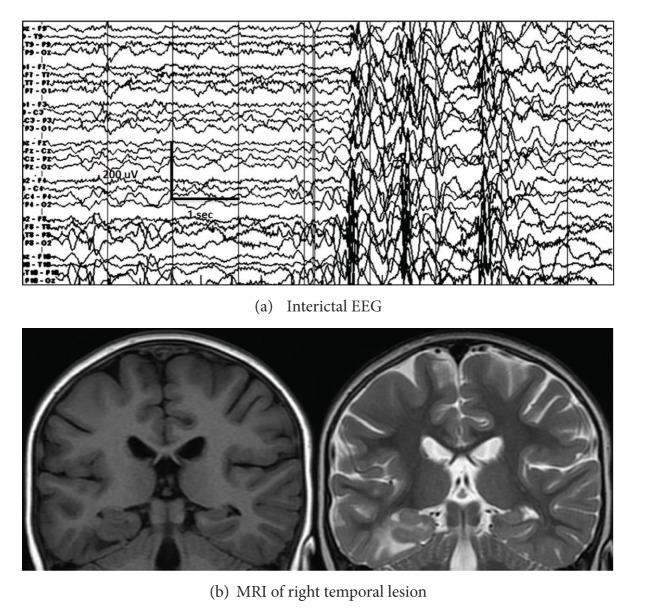
Asymmetric spasm: (a) interictal EEG showing synchronous generalized discharges consistently having maximal amplitude on the right, which corresponds to (b) right temporal focal lesion (WHO 1 ganglioglioma).

Variant patterns of classic hypsarrhythmia, including more organized or synchronous background, voltage lower than 500 *μ*V, and persistently focal features or asymmetries are often referred to as “modified hypsarrhythmia” but may occur in up to 1/3 of epileptic spasms [27, 28]. In partially treated spasms, especially in older children, the background may be more organized, synchronous, and of lower voltage (Figures 7 and 8). Persistent asymmetry of the ictal or interictal EEG suggests a focal lesion (Figure 9). However, the EEG and semiology may lack lateralizing or localizing features, despite focal structural etiology. Therefore, all children with intractable epileptic spasms due to structural etiology should be evaluated for possible resective surgery, regardless of the presence or absence of localizing EEG features [29, 30].

The EEG in children with CDKL5 initially demonstrates multifocal epileptiform discharges with gradual evolution to hypsarrhythmia or modified hypsarrhythmia, which persists well into early childhood. The prolonged duration of hypsarrhythmia is not typically seen in other causes of West syndrome [6, 31, 32].

The presence of prehypsarrhythmia patterns remains controversial. Retrospective analysis of serial EEGs prior to onset of hypsarrhythmia has demonstrated gradual evolution of multifocal potentially epileptogenic discharges with nearly normal background to frequent bihemispheric discharges with abnormal background [33]. Children who demonstrate worsening EEG background and an increase in bihemispheric epileptiform discharges should be considered at risk for developing epileptic spasms.

### 2.5. Dravet Syndrome

#### 2.5.1. Clinical Presentation

Dravet syndrome is also known as severe myoclonic epilepsy of infancy. Febrile status epilepticus manifests in previously normal infants prior to age 1 year either as generalized tonic-clonic seizures or a hemiconvulsion involving alternating sides. Afebrile seizures of multiple types then follow and are medically refractory. Head sensitivity, both to fever and ambient temperature, is a hallmark. Antiepileptic medications such as carbamazepine, oxcarbazepine, and phenytoin exacerbate seizures. Development stagnates and may regress. Intellectual disability, impulsivity, and ataxia emerges [34].

#### 2.5.2. Long-Term Prognosis

The prognosis for Dravet syndrome is typically poor although there is phenotypic variability. Frequent intractable seizures and recurrent refractory status epilepticus occur throughout childhood. Seizures often improve in adulthood, but nocturnal convulsive seizures continue [35]. Mortality is reported in up to 10%, and death often occurs due to seizure-related complications [36, 37]. 

#### 2.5.3. Etiologies

SCN1A mutations are found in 80% of patients with Dravet syndrome although mutations in SCN1B, SCN2A, and GABRG2 have also been reported. A minority of children with clinically diagnosed Dravet syndrome have no mutation found.

#### 2.5.4. Interictal EEG

The EEG findings in Dravet syndrome evolve with age. During the first year of life, the background EEG may be normal or show nonspecific slowing. Generalized or focal slowing may be present if the EEG is done after a prolonged seizure. The interictal epileptiform abnormalities increase between the second and fifth years of life and are typically generalized spike and polyspike and wave discharges. Multifocal or focal abnormalities are also present in the frontocentral or centrotemporal and vertex regions and likely represent fragments of diffuse discharges [38]. Photosensitivity and/or pattern sensitivity may be present in up to 40% of patients [34, 38]. In approximately 19–25% of children, generalized paroxysms and photosensitivity may decrease or disappear with age [39]. Focal and multifocal abnormalities may appear only during sleep [38].

#### 2.5.5. Ictal EEG

The ictal EEG findings depend on the seizure type. Pseudorhythmic spike and wave discharges with periodic attenuations can be present in hemiconvulsion or focal seizures with evolution to bilateral convulsive seizures [38]. Atypical absence seizures are seen within the first year after seizure onset and may be associated with eyelid myoclonus and generalized myoclonus. The EEG shows 2–4 Hz generalized spike wave discharges [40]. Myoclonic seizures can occur in clusters associated with generalized 3 Hz or faster spike wave with frontocentral predominance. Erratic, or segmental, myoclonia involving distal extremities and areas of the face, which are more palpable than visible, are epileptic seizures even though there is no EEG correlation [34]. Tonic seizures are rare in Dravet syndrome. The ictal EEGs in tonic seizures show a generalized fast rhythm lasting from 2 to 3 seconds.

## 3. Childhood Onset Epilepsy Syndromes

### 3.1. Electrical Status Epilepticus in Slow Wave Sleep Syndromes

#### 3.1.1. Clinical Presentation

Continuous spike wave in sleep syndrome (CSWS) and Landau Kleffner syndrome (LKS) both present in school-aged children with developmental and behavioral regression. Regression in CSWS is more global with significant executive dysfunction. A history of brain insult or abnormal development can be present before seizure onset and regression. Children with LKS are developmentally normal and then experience a regression in receptive language called acquired auditory agnosia.

#### 3.1.2. Long-Term Prognosis

With treatment, development and seizures improve although clinical and EEG relapses are common. As the child approaches adolescence, the seizures and EEG abnormalities in both CSWS and LKS spontaneously resolve. However, development does not normalize.

#### 3.1.3. Etiologies

Children with LKS have normal neuroimaging and normal development prior to onset of symptoms. There is no known cause of LKS. In contrast, children with CSWS are often found to have genetic, metabolic, or structural etiologies [41].

#### 3.1.4. Interictal EEG

 Both syndromes are associated with electrical status epilepticus in slow wave sleep (ESES) defined by nearly continuous epileptiform discharges during slow wave sleep. This EEG pattern must be present to make the diagnosis. 

**Figure 10:**
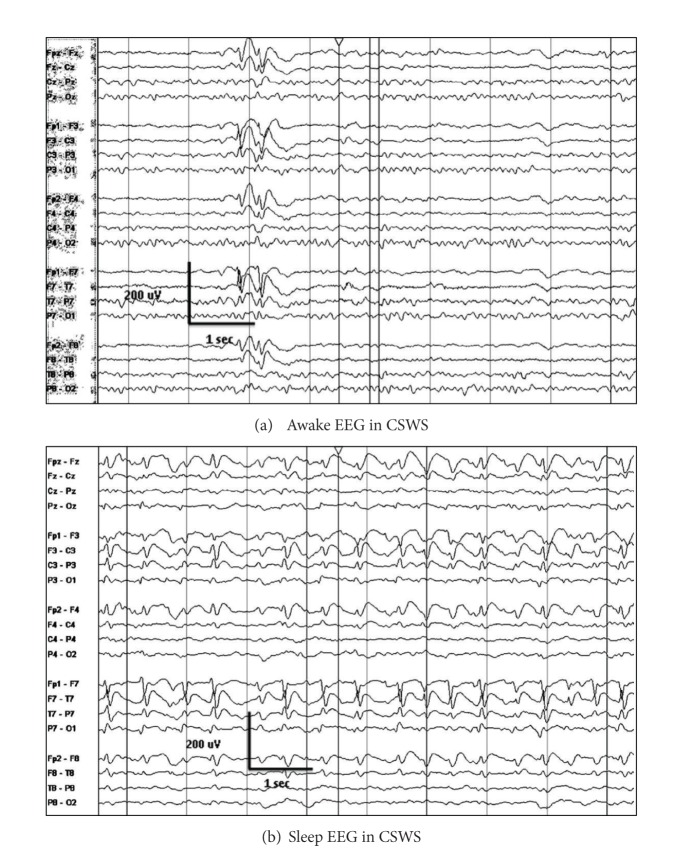
EEG in a patient with CSWS and history of left neonatal intraventricular hemorrhage: (a) awake interictal EEG in CSWS demonstrating generalized epileptiform discharges, maximal left, and (b) asleep interictal EEG demonstrating continuous epileptiform discharges maximally present over the frontocentral head regions, maximal left.

The EEG in CSWS demonstrates focal or diffuse slowing of the background with or without interictal discharges during wakefulness. Epileptiform discharges are focal, multifocal, or generalized. The epileptiform discharges in CSWS are often maximally present over the frontal regions, which may correlate with the observed executive dysfunction of these patients. There is significant activation if spike wave discharges during sleep, typically maximal over the frontal and frontocentral head regions. Furthermore, focal and multifocal discharges often have a broad distribution during sleep (Figure 10).

**Figure 11:**
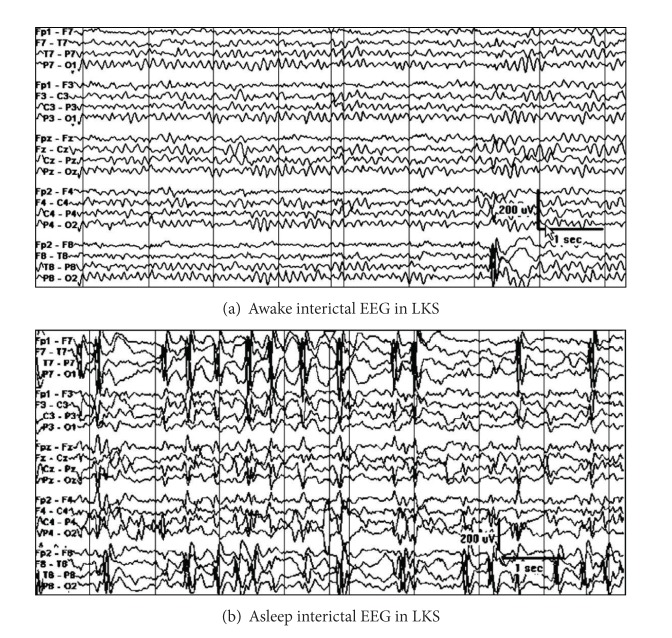
EEG in a nine-year old with LKS: (a) awake interictal EEG demonstrating infrequent potentially epileptiform discharges over the right temporal region and (b) asleep interictal EEG demonstrating nearly continuous discharges maximally present over the bilateral temporal regions.

The EEG in LKS during wakefulness may be normal or may demonstrate focal epileptiform discharges that are maximal over the frontotemporal or temporal regions. Like the children with CSWS, there may be focal or generalized slowing of the background activity. The EEG during sleep demonstrates nearly continuous spike wave discharges that are often maximal over the temporal regions (Figure 11) [41].

#### 3.1.5. Ictal EEG

Children with CSWS and LKS may or may not have seizures, making these unique epilepsy syndromes. The ictal EEG is similar to other focal seizures and is not diagnostic.

### 3.2. Lennox-Gastaut Syndrome

#### 3.2.1. Clinical Presentation

Lennox-Gastaut syndrome (LGS) manifests in children from 1 to 8 years of age. The most common seizures are tonic, atonic, and absence seizures. All seizure types may not be present simultaneously or at onset. The presence of other seizure types such as myoclonic seizures, unilateral clonic seizures, or focal seizures with or without evolution to bilateral convulsive seizures does not exclude LGS [42]. LGS can evolve from epileptic spasms. Developmental delay is present in 20–60% of patients at onset and increases to 75–95% after 5 years [43].

#### 3.2.2. Long-Term Prognosis

The long-term prognosis for Lennox-Gastaut syndrome is poor. Refractory seizures may improve with time but do not resolve. Recurrent status epilepticus also continues. Intellectual disability continues throughout life. Those with early onset epilepsy, especially those with a prior history of West syndrome, have the worst prognosis [44]. 

#### 3.2.3. Etiologies

Etiologies for LGS are heterogeneous. Up to 1/3 of children have no known cause of their epilepsy. Structural lesions are a common cause of LGS and may be due to congenital malformation so of cortical development, hypoxic-ischemic encephalopathy or other cerebrovascular events, and tuberous sclerosis complex. Genetic and metabolic disorders are also associated with LGS, but less frequently [44]. 

#### 3.2.4. Interictal EEG

**Figure 12:**
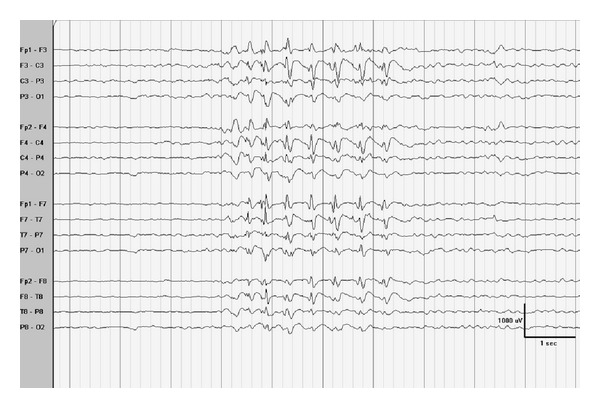
Slow spike and wave discharges in a four-year-old child with Lennox-Gastaut syndrome. Note the high amplitude of the generalized, anteriorly predominant, slow spike and wave discharges.

At seizure onset, the interictal EEG may demonstrate a slow and poorly organized background during wakefulness with normal EEG during sleep. The degree of slowing tends to correlate with severity of intellectual impairment. Diffuse slow spike wave discharges then occur in repetitive sequences but can be irregular infrequency, amplitude, morphology, and distribution. Slow spike wave discharges (SSW) consist of a spike (<70 ms) or a sharp wave (70–200 ms), followed by a positive deflection and a sinusoidal negative slow wave of 300–500 ms (Figure 12) [44]. SSWs repeat between 1 and 4 Hz, but typically at 1.5–2.5 Hz [45]. SSWs are abundant and at times prolonged without apparent clinical changes. Hyperventilation and photic stimulation do not generally increase SSW. Focal and multifocal epileptiform discharges are seen in 14–18% of patients [44].

In adulthood, the awake EEG may be normal or show generalized slowing. Generalized fast activity persists in sleep, but the SSW are present only in a minority of adults with LGS [46]. Many EEGs evolve to show independent multifocal spike wave discharges [47].

#### 3.2.5. Ictal EEG

**Figure 13:**
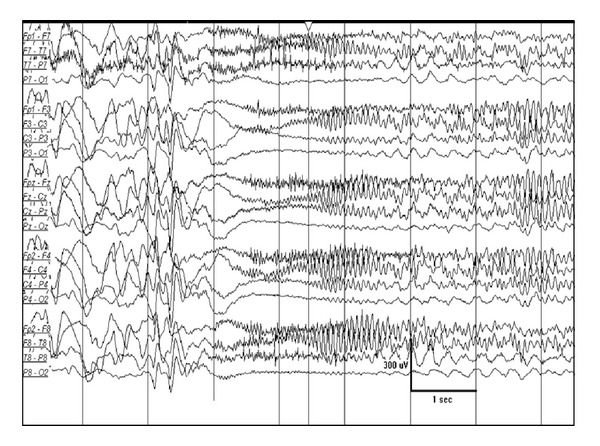
A 17-year-old man with history of autism spectrum disorder with a tonic seizure. Generalized voltage attenuation (electrodecremental pattern), bursts of low amplitude fast activity (15–25 Hz) with increasing amplitude from 50 to 100 *μ*V with a “recruiting” rhythm.

**Figure 14:**
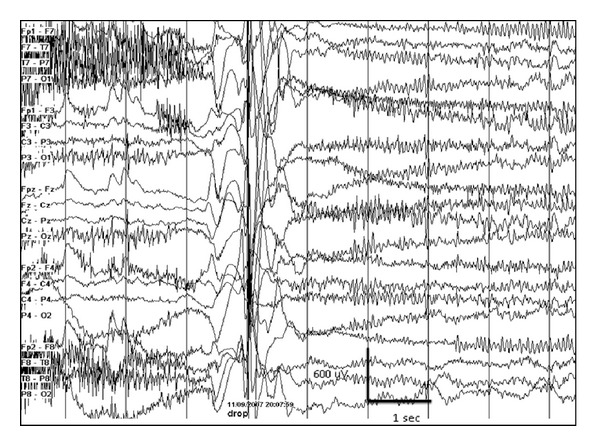
A 7-year-old girl with Lennox-Gastaut syndrome. Atonic seizure associated with generalized polyspike-and-wave, generalized voltage attenuation, and runs of low voltage fast activity.

The ictal EEG pattern depends on the recorded seizure type. Bursts of nearly continuous SSW may be associated with decreased responsiveness, representing atypical absence seizures. The ictal EEG shows high amplitude 1.5–2.5 Hz and is difficult to distinguish from the interictal pattern. Tonic seizures are associated with generalized voltage attenuation (electrodecremental pattern) or bursts of low-amplitude fast (15–25 Hz) activity with increasing amplitude 50–100 *μ*V (“recruiting” rhythm), followed by generalized delta slowing for several seconds before returning to baseline (Figure 13). Atonic seizures can be characterized by generalized spike-wave activity, generalized polyspike-and-wave activity, generalized voltage attenuation, or runs of low- or high- voltage fast activity (Figure 14). Myoclonic seizures may show generalized spike or polyspike and wave discharge corresponding to the myoclonic jerk. In adulthood, the tonic seizures and accompanying diffuse fast rhythms continue during sleep [48].

Status epilepticus occurs in more than 2/3 of all patients with LGS. Tonic status and atypical absence status are the most common. The EEG during status epilepticus may be difficult to appreciate from the abnormal interictal pattern of abundant SSW. Ictal spike wave discharges are more persistent and the EEG becomes more desynchronous and absence of posterior background activity [42]. 

### 3.3. Myoclonic-Atonic Epilepsy

#### 3.3.1. Clinical Presentation

Myoclonic-atonic epilepsy (MAE) is also known as myoclonic-astatic epilepsy, or Doose syndrome. Seizure onset is between 7 months and 6 years in previously normally developing children. It is more common in males by twofold, except in the first year of life during which the ratio is similar. Myoclonic-atonic seizures are characterized by initial vocalization or grunt, caused by quick contracture of the diaphragm, followed by atonic head or body drop. The presence of the myoclonic-atonic sequence accounts for 10% of children with MAE; 50% have isolated myoclonic seizures and 30% have isolated atonic seizures [49]. Other seizure types present include absence seizures, clonic seizures, and generalized tonic-clonic seizures. Tonic seizures are only rarely present. Status epilepticus of absence seizures and myoclonic-atonic seizures is common. 

#### 3.3.2. Long-Term Prognosis

The prognosis of MAE is variable. If effective treatment is found early, some children can become seizure-free and have a good cognitive outcome. Although prognosis cannot be predicted at initial presentation, those children with status epilepticus and cognitive decline have a poorer outcome. EEG features that suggest less favorable outcome include persistence of abnormal background slowing and failure of alpha rhythm to develop. 

#### 3.3.3. Etiologies

There is no known cause of MAE identified although there may be a possible genetic link to the generalized epilepsy with febrile seizures plus (GEFS+) family [50].

#### 3.3.4. Interictal EEG

The EEG is initially normal. Diffuse or focal theta activities have been described. Brief bursts of generalized polyspike and wave epileptiform activity at 2–5 Hz are noted. Occipital 4 Hz activity may also be seen and can be attenuated by eye opening [50]. Photoparoxysmal responses are associated with 3–7 Hz generalized spike and wave complexes. 

#### 3.3.5. Ictal EEG

All ictal EEGs demonstrate generalized spike or polyspike and wave complexes. Persistent focal EEG findings are not typically seen in MAE although apparent focal abnormalities or shifting laterality may be present and are likely fragments of generalized discharges [50]. 

## 4. Epilepsy Syndromes with Variable Age of Onset

### 4.1. Progressive Myoclonic Epilepsies

Progressive myoclonic epilepsies are rare disorders caused by metabolic, genetic, and neurodegenerative diseases. Children can present at all ages with multiple seizure types, worsening myoclonus, and developmental regression. Long-term prognosis and mortality depend on the specific etiology. The interictal EEG in the progressive myoclonic epilepsies demonstrates generalized slowing of the background with frequent generalized and multifocal epileptiform discharges. The myoclonus is associated with a generalized spike and wave discharge on EEG. Photosensitivity is common and can increase interictal and ictal discharges. These patterns are neither specific nor sensitive for determining specific etiology [51] although there are some clinical and neurophysiologic findings that can be helpful in determining etiology.

#### 4.1.1. Tay-Sachs

Tay-Sachs is due to hexosaminidase a deficiency and typically presents during infancy with exaggerated startle reflex to sound a developmental regression. The EEG can demonstrate fast spikes over the central head region [52].

#### 4.1.2. Myoclonic Epilepsy with Ragged Red Fibers

Mitochondrial disorders result in metabolic energy failure. The cerebral involvement manifests as seizures. Myoclonic epilepsy with ragged red fibers is an example of a mitochondrial disease that presents with multiple neurologic signs in addition to myoclonic epilepsy, including deafness, myopathy, optic atrophy, and cerebellar ataxia. The EEG is nonspecific, demonstrating slowing of the background with generalized and focal epileptiform discharges [51].

#### 4.1.3. POLG1 Mutation

**Figure 15:**
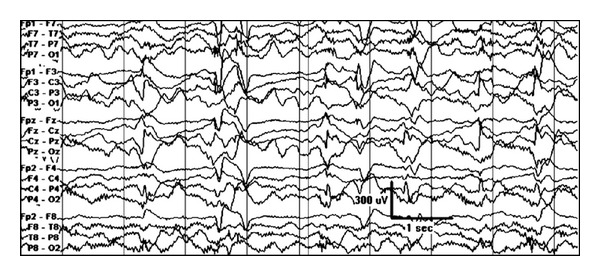
Interictal EEG in Alper's disease due to POLG1 mutation demonstrating high-amplitude generalized epileptiform discharges and suppressed background.

Mutation in the nuclear encoded mitochondrial POLG1 gene may manifest as Alpers disease or as ataxia syndromes such as myoclonus, epilepsy, myopathy, sensory ataxia (MEMSA) syndromes in older individuals. Alpers disease causes recurrent prolonged status epilepticus, hepatic failure, and cognitive decline. The EEG in Alpers disease demonstrates a slow or absent posterior dominant rhythm with multifocal and generalized epileptiform activity [53] (Figure 15). Status epilepticus is common and may have an EEG correlation of high amplitude delta activity that is maximal over the posterior head regions with superimposed epileptiform activity [54].

#### 4.1.4. Lafora Disease

During adolescence, Lafora disease causes recurrent seizures and cognitive regression. Early in the course of Lafora disease, the generalized epileptiform discharges occur at a frequency of approximately 3 Hz. As the disease progresses, there is slowing of the background and the frequency of the discharges increases from 3 Hz up to 6–12 Hz [51].

#### 4.1.5. Unverricht-Lundborg Disease

Similar to Lafora disease, Unverricht-Lundborg disease is also associated with adolescent onset epilepsy with cognitive regression. The EEG in Unverricht-Lundborg disease is nonspecific during wakefulness, showing the expected generalized slowing, epileptiform discharges, and photoparoxysmal response. However, the sleep EEG patterns remain essentially normal, which is not a typical finding in other progressive myoclonic epilepsies [51].

#### 4.1.6. Neuronal Ceroid Lipofuscinosis

 Neuronal ceroid lipofuscinosis (NCL) also presents in adolescence with refractory seizures and regression, as well as visual loss. As in other storage diseases, there is progressive slowing of the background activity on the EEG, leading to loss or “vanishing” of EEG activity in NCL [52]. Photosensitivity with a photoparoxysmal response below 3 Hz may also be present [55].

### 4.2. Immune-Mediated Syndromes

Autoimmune-mediated etiologies for epilepsy are increasingly recognized and should be suspected in patients with multifocal neurologic signs and symptoms, including intractable epilepsy, psychiatric disorder, cognitive dysfunction, movement disorders, sleep disturbance, or autonomic dysfunction with acute or subacute onset and a progressive course. There may be personal or family history of autoimmunity or symptom onset after illness or immunizations.

#### 4.2.1. Rasmussen's Encephalitis

**Figure 16:**
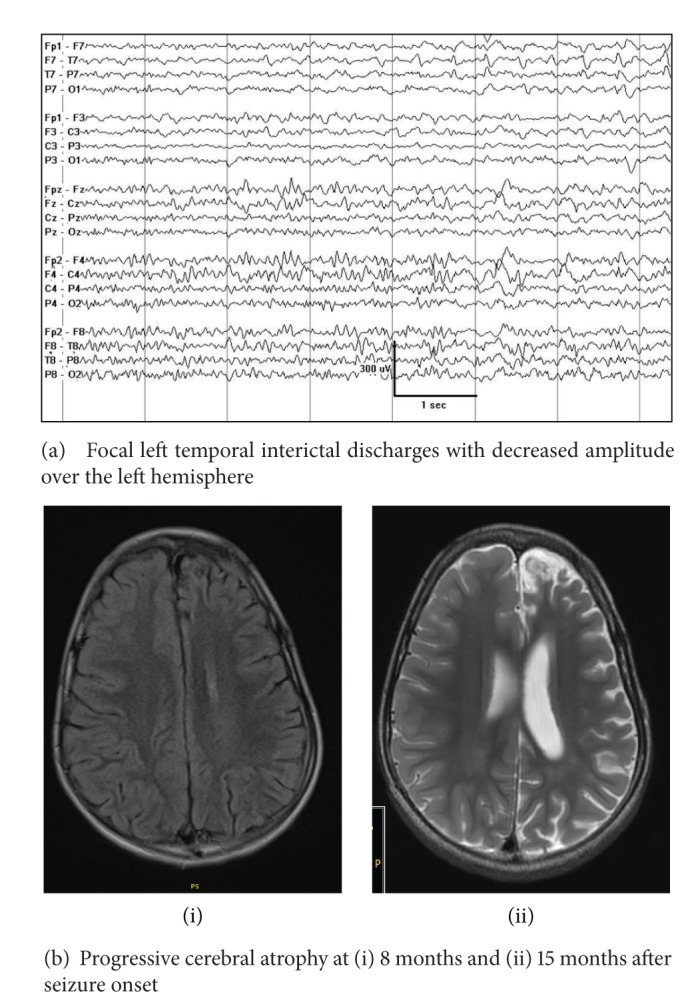
EEG in a previously well child presenting with epilepsia partialis continua, progressive hemiparesis, and contralateral cerebral atrophy, consistent with Rasmussen's syndrome.

Rasmussen's encephalitis is an autoimmune-mediated progressive focal epilepsy with associated increasing contralateral hemiatrophy. The EEG may show lateralized background slowing ipsilateral to the hemiatrophy. Epilepsia partialis continua is common and may not have an EEG correlation (Figure 16).

#### 4.2.2. Voltage-Gated Potassium Channel Complex Antibodies

Voltage-gated potassium channel (VGKC) complex antibodies are associated with intractable epilepsy and encephalopathy in children. LGI1 and CASPR2 are the two most commonly identified VGKC target antigens. The interictal EEG demonstrates nonspecific slowing of the background and multifocal or generalized epileptiform discharges. Faciobrachial dystonic seizures are characterized by brief unilateral facial grimace with concurrent ipsilateral arm dystonia that occurs many times per day and is often seen in children and adults with VGKC-related encephalopathy [56]. The ictal EEG demonstrates contralateral rhythmic frontotemporal spike wave discharges. 

#### 4.2.3. NMDA Receptor Antibodies

NMDA receptor (NMDAR) antibodies are also associated with intractable epilepsy and encephalopathy in children. The EEG in patients with NMDAR antibodies demonstrates diffuse nonspecific slowing but often does not reveal potentially epileptogenic discharges. In a minority of patients, nearly continuous 1–3 Hz delta activity with superimposed bursts of beta frequency fast activity is present, termed “extreme delta brush” because it is reminiscent of the spindle delta brush pattern seen in premature infants [57]. 

### 4.3. Specific EEG Findings in Other Childhood Developmental Disabilities

Rarely, neurodevelopmental syndromes are associated with specific EEG patterns, both epileptiform and nonepileptiform. Recognition of these specific EEG patterns can be helpful in identifying the underlying syndrome.

#### 4.3.1. Angelman Syndrome

**Figure 17:**
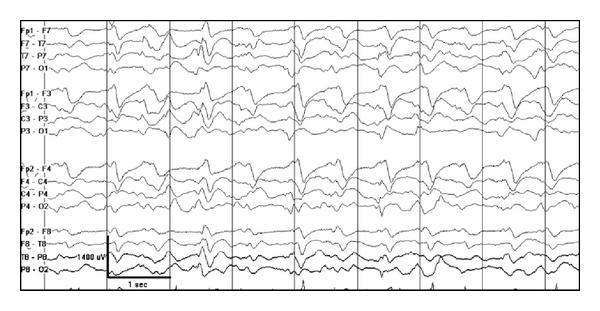
A 5 year-old girl with Angelman syndrome. The notched-delta pattern, a variant of ill-defined slow spike-and-wave complexes, in which spikes are superimposed on the ascending or the descending phase of the slow wave giving it a notched appearance.

 Angelman syndrome is a neurodevelopmental disorder caused by absence of functional maternally inherited UBE3A gene on chromosome 15q11–q13 [58]. Children have severe developmental delay, poor, or no language acquisition, happy demeanor, stereotypies, and wide-based gait. Epilepsy and EEG abnormalities are seen in 80% of patients [59]. Generalized diffuse slowing with normal sleep patterns is seen. The notched delta pattern (Figure 17) is seen in 73% of patients with Angelman syndrome at a mean age of 5.2 years [60]. Interictal epileptiform abnormalities are focal or multifocal in distribution.

#### 4.3.2. Lissencephaly

**Figure 18:**
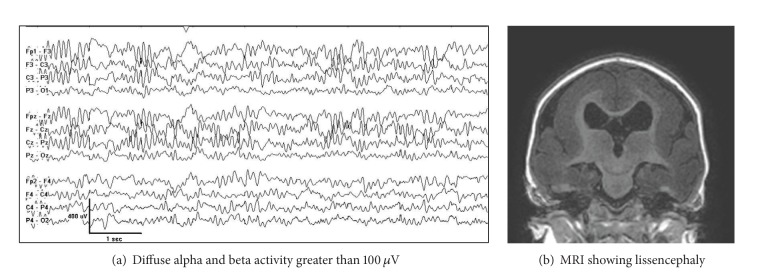
Lissencephaly due to *DCX *mutation in a 6 month-old male.

Lissencephaly is a severe malformation of cortical development with agyria or pachygyria. Onset of epileptic spasms may be the presenting feature that leads to diagnosis. The background EEG is nonspecific. Three reported EEG patterns are seen in lissencephaly: (1) diffuse greater than 100 *μ*V alpha and beta activity in all cortical regions (Figure 18), (2) alternating high amplitude >300 *μ*V bursts of sharp and slow waves followed by short periods of attenuation, and (3) high amplitude spike wave or sharp wave activity, without alpha or beta frequencies or attenuations. Children with anterior agyria-pachygyria with DCX mutation have diffuse moderate amplitude alpha activity, whereas those with posterior predominant cortical abnormality seen in LIS1 mutation tend to have the EEG pattern with bursts of sharp and slow waves with periods of attenuation.

#### 4.3.3. Subacute Sclerosis Panencephalitis

**Figure 19:**
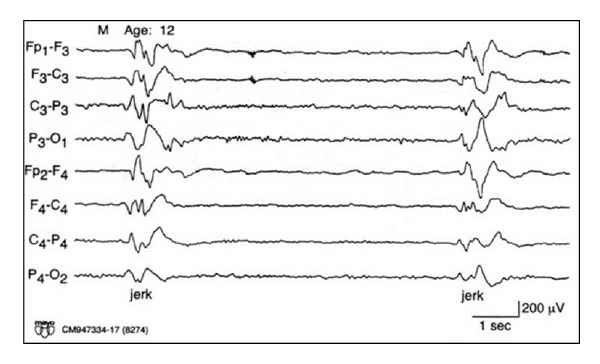
A 12-year-old boy with subacute sclerosis panencephalitis. Note high voltage, polyphasic sharp-and slow-wave complexes, lasting from 0.5 to 2 seconds in a pseudoperiodic pattern.

Subacute sclerosis panencephalitis (SSPE) is a postmeasles encephalitis causing a degenerative process in children and adolescents. It is characterized by motor jerks and progressive intellectual deterioration. The typical EEG pattern consists of high voltage, polyphasic sharp- and slow-wave complexes, lasting from 0.5 to 2 seconds in duration and occurring repetitively in a pseudoperiodic fashion (Figure 19). The complexes are usually generalized and bisynchronous but may be asymmetric or occur in a more lateralized or focal fashion. The complexes may occur at irregular intervals initially. As the disease progresses, the complexes occur at regular intervals, typically every 4 to 15 seconds but may range up to 1 to 5 minutes. Stereotyped motor jerks or spasms occur in association with the periodic complexes. The movements may take place simultaneously with, prior to, or following the periodic complexes. The slow-wave complexes occurring in a regular and periodic fashion and having a constant relationship to motor movements make this pattern specific to SSPE [61].

## 5. Conclusion 

When children present with new onset seizures, the long-term outcome is of great concern to parents and clinicians. Knowledge of the EEG findings supportive of specific electroclinical syndromes is important for accurate diagnosis. Classification of epilepsy electroclinical syndromes is essential particularly in epileptic encephalopathy to guide optimal treatments and counsel family regarding expected outcome.

## Figures and Tables

**Table 1:** Summary of clinical characteristics and EEG features at presentation in early and childhood onset epileptic encephalopathy.

Epilepsy syndrome	Clinical features				EEG features at presentation		
	Age of seizure onset	Seizure types	Underlying etiology	Prognosis	Background	Interictal	Ictal
Early infantile epileptic encephalopathy (ohtahara syndrome)	First 2 weeks of life	Tonic seizures	Cerebral structural abnormality, genetic abnormalities (i.e., *STXBP1*)	25% die by 2 years or evolves to West syndrome and profound disability	Suppression burst pattern in awake and sleep	High voltage (150–350 uV) paroxysm	Generalized paroxysms or focal discharges

Early myoclonic encephalopathy	First weeks of life	Myoclonic seizures (erratic/fragmentary/ generalized); focal seizures	Metabolic genetic etiologies (nonketotic hyperglycinemia, pyridoxine/pyridoxal-5-phosphate dependency, molybdenum cofactor deficiency, organic aciduria, amino-acidopathies)	50% die within first year or profound disability	Suppression burst pattern, enhanced by sleep	High voltage (150–350 uV) paroxysm	Generalized paroxysms or focal discharges

Migrating focal seizures in infancy	3 months	Focal motor seizures with autonomic manifestations	Unknown; SCN1A, PLCB1, KCNT1 mutations; 2q24, 16p11.2 copy number variants	High mortality before 1 year or profound disability (cortical visual impairment; acquired microcephaly)	Hemispheric background slowing	Multifocal discharges, maximal in temporal and rolandic regions	Rhythmic, monomorphic alpha or theta discharges in noncontiguous brain regions

West syndrome	3–8 months	Epileptic spasms	Heterogenous (congenital cortical malformations, tuberous sclerosis, trisomy 21, trisomy 18, CDKL5, ARX, MECP2)	Depends on etiology; other seizure types evolve by about 5 years	Poorly organized, high amplitude (500–1000 mV), generalized slowing	Multifocal epileptiform discharges with generalized electrodecrement	Generalized sharp wave followed by electrodecrement

Dravet syndrome	6 months	Febrile status epilepticus, alternating hemiconvulsions→ absence, and myoclonic seizures	80% SCN1A mutation	Mortality in childhood 10%, intellectual disability, or crouched gait without spasticity in adults	Normal, generalized or focal slowing	Generalized, multifocal or focal discharges; photoparoxysmal response	Generalized paroxysms or focal discharges

ESES-related syndromes	5–8 years	Focal seizures	CSWS structural; LKS unknown	Relapsing-remitting course or age limiting by teenage years	Normal or focal/diffuse slowing	Focal/multifocal/generalized discharges; marked sleep activation with increased interictal spatial distribution or bilateral synchrony; sleep spike wave index >85%; CSWS-frontal predominant LKS-temporal predominant	Focal discharges

Lennox-Gastaut syndrome	1–8 years	Multiple (tonic, atonic, absences, myoclonic, or focal)	Heterogenous	Intellectual disability	Normal or generalized slowing	Frequent slow spike waves 1.5–2.5 Hz or multifocal	Absence-low spike and waves; tonic-generalized attenuation with recruiting rhythm; atonic-generalized polyspike/spike waves, or attenuation; myoclonic-generalized polyspike/spike waves

Myoclonic-atonic epilepsy	7 months–6 years	Multiple (atonic, myoclonic, absences, or rarely tonic)	No consistent etiology	50% normal cognition at last followup	Normal or mild diffuse/focal slowing	Generalized polyspike-and-wave discharges; photoparoxysmal response	Generalized spike or polyspike-and-wave

Progressive myoclonic epilepsies	Varies by etiology	Prominent myoclonic seizures	Inborn errors of metabolism and mitochondrial disorders (Tay-Sachs, Alpers syndrome, Lafora disease, Unverricht-Lundborg disease, or neuronal ceroid lipofuscinosis)	Developmental regression/dementia; mortality depends on etiology	Generalized slowing	Generalized/multifocal discharges; photoparoxysmal response in Unverricht-Lundborg and neuronal ceroid lipofuscinosis	Generalized discharges

ESES: electrical status epilepticus in slow wave sleep; CSWS: continuous spike wave in sleep; LKS: Landau-Kleffner syndrome.
